# Integrating multi-modal omics to identify therapeutic atherosclerosis pathways for coronary heart disease

**DOI:** 10.1016/j.ebiom.2025.105966

**Published:** 2025-10-15

**Authors:** Sophie C. de Ruiter, Marion van Vugt, Chris Finan, Diederick E. Grobbee, Dominique P.V. de Kleijn, Gerard Pasterkamp, Hester M. den Ruijter, Ernest Diez Benavente, Sanne A.E. Peters, A. Floriaan Schmidt

**Affiliations:** a Julius Center for Health Sciences and Primary Care, University Medical Center Utrecht, Utrecht University, Utrecht, the Netherlands; b Department of Cardiology, Amsterdam Cardiovascular Sciences, Amsterdam University Medical Centres, University of Amsterdam, Amsterdam, the Netherlands; c Faculty of Population Health, Institute of Cardiovascular Science, University College London, London, United Kingdom; d Division of Heart and Lungs, Department of Cardiology, University Medical Center Utrecht, Utrecht University, Utrecht, the Netherlands; e UCL British Heart Foundation Research Accelerator, London, United Kingdom; f Health Data Research UK and Institute of Health Informatics, University College London, London, United Kingdom; g Department of Vascular Surgery, University Medical Center Utrecht, Utrecht University, Utrecht, the Netherlands; h Central Diagnostic Laboratory, University Medical Center Utrecht, Utrecht University, Utrecht, the Netherlands; i Laboratory of Experimental Cardiology, Division of Heart and Lungs, University Medical Center Utrecht, Utrecht University, Utrecht, the Netherlands; j The George Institute for Global Health, School of Public Health, Imperial College London, London, UK

**Keywords:** Coronary heart disease, Atherosclerosis, Drug targets, Metabolism pathways, Mendelian randomisation, Single-cell RNA sequencing

## Abstract

**Background:**

Urinary metabolism breakdown products reflect metabolic changes in atherosclerosis-relevant tissues and may contain relevant therapeutic leads. We integrated data on urinary metabolism breakdown products, plasma proteins, atherosclerotic plaque tissue, and single-cell expression to identify druggable metabolic pathways for coronary heart disease (CHD).

**Methods:**

Mendelian randomisation was employed to interrogate findings from independent genome-wide association studies on 954 urinary metabolism breakdown products, 1562 unique proteins, and 181,522 CHD cases, establishing directionally concordant associations. Using the Athero-Express Biobank, concordant plasma proteins were linked to plaque vulnerability using protein and mRNA expression in plaque. Single-cell RNA sequencing data obtained from carotid plaque samples were used to test for differential expression of concordant proteins across plaque cell types.

**Findings:**

In total, 29 urinary metabolism breakdown products associated with CHD, predominantly originating from amino acid metabolism (n = 12) or unclassified origin (n = 9). We identified 113 plasma proteins with directionally concordant associations with these urinary metabolism breakdown products and CHD. Of the 110 proteins available in plaque, 16 were associated with plaque vulnerability. This included positive control proteins targeted by drugs indicated for CHD, such IL6R (targeted by tocilizumab) and AT1B2 (targeted by digoxin), as well as a potential repurposing opportunity C1S (targeted by sutimlimab).

**Interpretation:**

We have identified amino acid metabolism as an important contributing pathway to CHD risk. These metabolism pathways were linked to 16 prioritised proteins relevant for CHD with involvement in atherosclerotic plaques, providing important insights for drug development.

**Funding:**

SR and SP are supported by a VIDI Fellowship (project number 09150172010050) from the Dutch Organisation for Health Research and Development (ZonMW) awarded to SP. AFS is supported by 10.13039/501100000274BHF grant PG/22/10989, the UCL BHF Research Accelerator AA/18/6/34223, the UCL BHF Centre of Research Excellence RE/24/130013, MR/V033867/1, the National Institute for Health and Care Research University College London Hospitals Biomedical Research Centre, and the EU Horizon scheme (AI4HF 101080430 and DataTools4Heart 101057849). MV is supported by a postdoc talent grant from the 10.13039/100019741Amsterdam Cardiovascular Sciences. This work was funded by 10.13039/100014013UK Research and Innovation (UKRI) under the UK government's Horizon Europe funding guarantee EP/Z000211/1, and by the 10.13039/501100000833Rosetrees CF-2-2023-M-2/122. This publication is part of the project “Computational medicine for cardiac disease” with file number 2023.022 of the research programme “Computing Time on National Computer Facilities” which is (partly) financed by the Dutch Research Council (NWO).

**Research in context**

**Evidence before this study**

We searched PubMed for ("coronary heart disease"[All Fields] OR "coronary artery disease"[All Fields] OR "atherosclerosis"[All Fields] OR CHD[All Fields] OR CAD[All Fields]) AND (metabolomics[All Fields] OR "urinary metabolites"[All Fields] OR "metabolic breakdown products"[All Fields] OR "metabolism breakdown products"[All Fields]) AND (proteomics[All Fields] OR "plasma proteins"[All Fields]) for articles published up to July 5, 2025 without any language restriction, to identify studies that used an integration of proteomics and metabolomics in the context of coronary heart disease. This search identified 43 studies, of which 6 used at least two layers of omics data. One of these studies integrated proteomics with metabolomics for atherosclerosis classification, but results were only based on plasma protein expression. No study integrated data from different layers of omics to prioritise potential therapeutic targets, with a validation in carotid plaque tissue.

Previous research has shown that altered metabolism is closely related to immune response and can contribute to the progression of atherosclerosis and coronary heart disease (CHD). Studies have demonstrated associations between metabolic pathways and cardiovascular disease outcomes, however there have not been any systematic attempts at a comprehensive multi-omics approach to identify metabolic breakdown products involved in CHD progression and their links to CHD-related protein expression which could serve as drug targets.

**Added value of this study**

This study integrates multi-modal data sources, including large-scale genetic data on 954 urinary metabolic breakdown products, on 1562 plasma proteins and on CHD, as well as protein measurements obtained from carotid plaques from patients at high risk of a cardiovascular event. We aimed to identify metabolic origins linked to CHD as well as proteins relevant for therapeutic treatment. We identified 16 proteins that were expressed both in plasma and carotid plaques, associated with CHD risk and plaque vulnerability, and were related to five upstream metabolic pathways altered in CHD. For example, the protein C1S, targeted by drugs indicated for anaemia, angioedema and immune system disorders, was related to breakdown products of xenobiotic metabolism and associated with an increased CHD risk as well as higher plaque vulnerability, presenting an opportunity for drug repurposing. Additionally, we identified known therapeutic targets, such as CAH1 (targeted by acetazolamide indicated for heart failure). This study provides a set of prioritised plasma proteins that may serve as relevant therapeutic targets within CHD management.

**Implications of all the available evidence**

Our findings are relevant for both clinical practice and drug development. By linking CHD-associated plasma proteins to CHD-associated metabolic pathways, this study provides potential targets for therapeutic intervention. The identification of known drug targets supports the validity of our approach, while the discovery of other protein targets suggests potential for therapeutic treatment. Furthermore, the results highlight the potential for drug repurposing, exemplified by C1S, which is currently targeted by drugs for anaemia, angioedema, and immune disorders but may also be relevant in CHD treatment. These insights contribute to a better understanding of disease mechanisms and offer opportunities for developing metabolic pathway-based interventions to reduce CHD risk and improve patient outcomes.

## Introduction

Atherosclerosis is characterised by the development of lesions accumulating lipoproteins and inflammatory cells in the arterial wall. As atherosclerosis progresses, plaque accumulates and may progress into high-risk vulnerable plaques. Such plaques are characterised by a thin fibrous cap, a large lipid core and high levels of inflammatory cells,^1^^,^^2^ making them particularly prone to rupture. Plaque rupture can lead to rapid thrombus formation blocking the blood flow to the heart muscle, potentially leading to acute clinical events such as coronary heart disease (CHD), which remains a leading cause of burden and death globally, accounting for around 9 million deaths annually worldwide.^3^

Metabolic disorders, such as metabolic syndrome (the co-occurrence of hypertension, hypercholesterolaemia, diabetes, and obesity), are associated with a metabolic, pro-inflammatory, and pro-thrombotic state which are key drivers of atherosclerosis,^4^ reflecting the close interrelationship between immune response and metabolic homoeostasis, where dysfunction in one system can adversely affect the other.^5^ Plasma proteins are markers of disease and targets of most drug compounds. Previous research has already linked plaque phenotypes to circulating proteins, identifying reduced plasminogen activator inhibitor levels in patients with an intermediate coronary artery disease risk profile, for example.^6^

Urinary metabolism breakdown products, which are the end-products of metabolism excreted in urine, reflect metabolic changes occurring in atherosclerosis-relevant tissues (e.g., body fat, liver, the arterial wall). Although these breakdown products are downstream and not directly actionable as they have been excreted from the body, they provide a non-invasive measure of upstream metabolic alterations that may contribute to CHD development. Given the physiological interplay between cardiac and renal function—such as through the renin-angiotensin-aldosterone system and the pathophysiology of cardiorenal syndrome—urinary breakdown products may reflect systemic metabolic disruptions originating in tissues relevant for cardiovascular health. In contrast, plasma proteins are both reflective of biological activity and actionable targets for therapeutic intervention. Therefore, integrating data on urinary metabolism breakdown products and plasma proteins enables the identification of relevant metabolic pathways and potential drug targets, offering a comprehensive approach to understanding and intervening on the progression of CHD.

In the current study, we used Mendelian randomisation (MR) to identify metabolic pathways for CHD by leveraging genetic data on urinary metabolism breakdown products (954 urinary metabolism breakdown products), plasma proteins (1562 unique plasma proteins), and CHD (181,522 cases). The fixed nature of genotypes, established during gametogenesis^7^^,^^8^ allows genetic variants to be used as instrumental variables in MR, which is therefore robust against the presence of confounding bias and potential reverse causation. In the context of cardiovascular disease, this approach has been empirically validated.^9^, ^10^, ^11^, ^12^ Plaque involvement was established using data from patients undergoing carotid endarterectomy and participating in the Athero-Express (AE) Biobank on protein expression, mRNA expression, and single-cell RNA sequencing. Finally, the potential druggability of these prioritised proteins was evaluated through linkage to ChEMBL.

## Methods

### Genetic data

Genetic associations with 954 urinary metabolism breakdown product values were available from a genome-wide association study (GWAS) conducted on 1627 individuals (55% men and mean age 63.5 years with standard deviation of 9.97 years) using a non-targeted mass spectrometry Metabolon assay.^13^ This approach allows for comprehensive detection of breakdown products, including those of unknown origin. Metabolism breakdown products were grouped based on their metabolic origins, which led to nine metabolism classes: amino acid metabolism, carbohydrate metabolism, cofactor and vitamin metabolism, energy metabolism, lipid metabolism, nucleotide metabolism, peptide metabolism, xenobiotic metabolism, or unclassified metabolism. Metabolism breakdown products with an origin in xenobiotic metabolism are breakdown products from substances not naturally produced by the human body, such as drugs, pollutants, and synthetic compounds. Unclassified metabolism breakdown products represent potential biomarkers, as they have not yet been related to an established metabolic origin. These breakdown products were noted for their recurring chromatographic and spectral properties and have assigned unique COMP-IDs and/or CHEM-IDs, along with their mass-to-charge (*m*/*z*) ratio and retention index values (Appendix Table S1). These identifiers help improve reproducibility and enable consistent detection across platforms, even for compounds with currently unclassified metabolic origin.

We used eight GWAS on genetic associations with plasma protein values, detailed in the Supplemental Methods, and a GWAS on genetic associations with incident or prevalent CHD (181,522 cases among 1,165,690 participants from European ancestry) obtained from Aragam et al.^14^

### Mendelian randomisation analyses

**Fig. 1:**
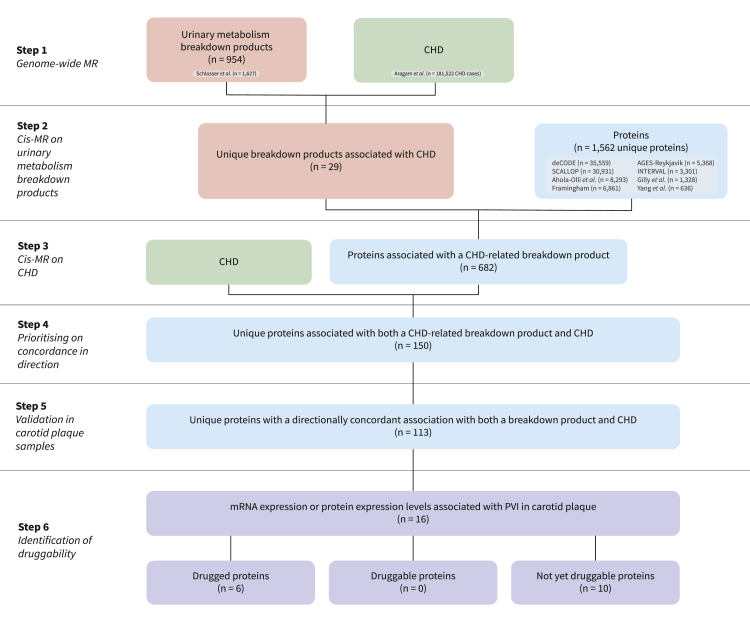
**Flowchart of the main steps of this study.** N.B. The first three steps are conducted using Mendelian randomisation, based on indicated GWAS data for CHD, urinary metabolism breakdown products, and plasma proteins. Directionally concordance is explained in Fig. 2 and the Methods section. Measurements on mRNA and protein expression are available from carotid plaque samples from the Athero-Express Biobank. Druggability of plasma proteins is determined using ChEMBL and the British National Formulary. Abbreviations: CHD = coronary heart disease, MR = Mendelian randomisation, PVI = plaque vulnerability index.

Three MR analyses were conducted: 1) genome-wide MR to identify urinary metabolism breakdown products associated with CHD through upstream pathways (step 1 in Fig. 1, Appendix Table S1), 2) *cis*-MR to identify plasma proteins affecting values of urinary metabolism breakdown products (step 2 in Fig. 1), and 3) *cis*-MR to identify plasma proteins associated with CHD (step 3 in Fig. 1). For the genome-wide MR, genetic variants were selected from across the genome, while the *cis*-MR utilised genetic variants selected from a 200 kilobase pair window around and within the protein encoding gene. In all MRs, variants were selected based on an exposure F-statistic of at least 24 (reducing the risk of weak instrument bias) and a minimal minor allele frequency of 0.01. The selected variants were clumped to a linkage disequilibrium (LD) r-squared of 0.30, based on a random sample of 5000 unrelated UK Biobank participants.^15^ The *cis*-MR analyses (steps 2–3 in Fig. 1) were conducted per individual protein GWAS, where the largest sample size GWAS was used if protein measurements overlapped in different studies.

MR analyses were conducted using generalised least squares (GLS) implementations of the inverse variance weighted (IVW) estimator, as well as MR-Egger estimator, known for its robustness to pleiotropy.^16^ To ensure sufficient instrument strength, we included only variants with an F-statistic ≥24. The GLS implementation was used to additionally correct for residual LD, which optimised estimator precision.^17^ To reduce the potential for horizontal pleiotropy, variants with large leverage statistics (larger than three times the mean leverage) and/or outlier statistics (Chi-square larger than 10.83) were excluded. We discarded analyses with fewer than six variants, ensuring we had sufficient data to accurately model the exposure effects. To further minimise the potential of horizontal pleiotropy, we applied a model selection framework identifying the MR model (IVW or MR-Egger) that is most supported by the available data.^18^ While MR-Egger allows for directional pleiotropy, we note that the intercept test is generally underpowered when few variants are available^16^ and therefore evaluate potential bias due to remaining horizontal pleiotropy using the more powerful Q-test. By comparing models using criteria such as the goodness-of-fit and parsimony, the model selection framework we used determines whether the additional parameter of MR-Egger (which accounts for directional pleiotropy) is supported by the data, or whether the more parsimonious IVW model is preferable. This model selection process reduces the risk of overfitting and susceptibility to bias from pleiotropic instruments.

### Triangulation of evidence through consideration of concordant CHD effects

**Fig. 2:**
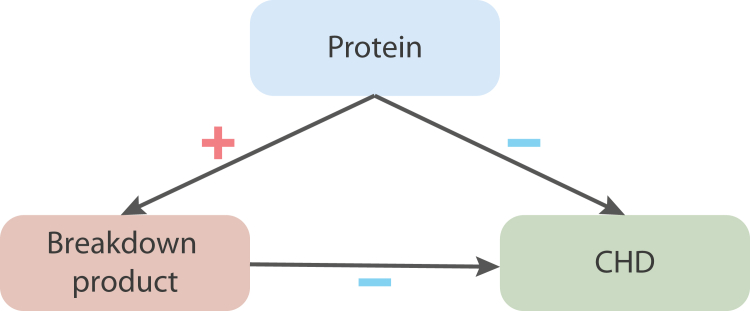
**Example of a triangulated association with directional concordance between a protein and its effects on urinary metabolism breakdown product values and CHD.** N.B. The plus symbol (in red) represents a positive association. The minus symbol (in blue) represents a negative association (a risk-decreasing effect). Directional concordance is achieved when the direction of effects aligns consistently, whether increasing or decreasing.

We integrated findings from the three distinct MR analyses to triangulate evidence on the effects of urinary metabolism breakdown products and proteins on CHD. This involved 1) identifying urinary metabolism breakdown products affecting CHD, 2) identifying proteins associated with these urinary metabolism breakdown products, and 3) focussing on the subset of proteins with an effect on CHD that was directionally concordant with the effect of a urinary metabolism breakdown product on CHD and the protein on the same breakdown product. For example, if a higher value of a urinary metabolism breakdown product reduced CHD risk, and a protein increased the value of this urinary breakdown product, the protein effect was considered directionally concordant if higher protein values reduced CHD risk. We describe concordance in direction as a triangulated association (Fig. 2). Given that potential horizontal pleiotropy acts through distinct pathways in each analysis (e.g., pre-translational pleiotropy in *cis*-MR versus pre- or post-translational horizontal pleiotropy in genome-wide MR^19^), focussing on results with directional concordance ensures identification of a robust subset of results with likely limited residual bias due to horizontal pleiotropy.

### Athero-Express Biobank atherosclerotic plaques

Next, the Athero-Express (AE) Biobank data was used to prioritise findings on atherosclerotic plaque involvement. The AE Biobank is a prospective cohort study of patients undergoing carotid endarterectomy in the Netherlands and is one of the largest biobanks worldwide with detailed plaque phenotyping. Patients enrolled in the AE Biobank were at risk of CHD, as well as other atherosclerosis associated diseases such as ischaemic stroke, with carotid plaques from these patients providing a relevant marker of generalised atherosclerosis.^20^, ^21^, ^22^, ^23^, ^24^ Specifically, the AE contains samples of patients undergoing carotid endarterectomy, where we identified potential associations between protein (194 patients) or mRNA (632 patients) expression in plaque and the plaque vulnerability index. In addition, single-cell RNA sequencing data (4948 cells and 46 patients) were analysed to study cellular plaque expression. Please see Supplemental Methods for the relevant details, and Table 1 for characteristics of patients participating in the AE Biobank.

**Table 1:** Characteristics of Athero-Express Biobank participants for which protein expression and mRNA expression data was available.

	Protein expression (n = 194)	mRNA expression (n = 632)
Age, years	69.2 (8.6)	68.4 (8.9)
Male	145 (74.7%)	476 (75.3%)
Hypertension	167 (86.1%)	447 (72.2%)
Diabetes	44 (22.7%)	136 (21.5%)
Current smoker	69 (35.6%)	226 (36.2%)
Former smoker	91 (46.9%)	283 (44.8%)
BMI, kg/m^2^	26.9 (4.2)	26.6 (3.8)
Systolic blood pressure, mmHg	153.6 (24.8)	154.3 (25)
Diastolic blood pressure, mmHg	81.9 (12.3)	82.5 (13.6)
Total cholesterol, mmol/L	4.5 [3.7, 5.3]	4.6 [3.8, 5.5]
LDL cholesterol, mmol/L	2.6 [1.9, 3.3]	2.6 [2, 3.4]
HDL cholesterol, mmol/L	1.1 [0.8, 1.4]	1.1 [0.9, 1.3]
Triglycerides, mmol/L	1.2 [1.0, 1.7]	1.4 [1, 2]
Antihypertensive drug use	149 (76.8%)	488 (77.3%)
Lipid lowering drug use	146 (75.3%)	480 (76%)
Anticoagulant drug use	26 (13.4%)	76 (12%)
Antiplatelet drug use	170 (87.6%)	564 (89.4%)

N.B. Characteristics are presented as mean (standard deviation), median [25th, 75th percentile], or number (percentage of total population).

Abbreviations: BMI, body mass index; LDL, low-density lipoprotein; HDL, high-density lipoprotein.

See for more information Mokry et al. (2022).^6^

### Associations with plaque vulnerability

We tested for associations between both protein expression and mRNA expression levels of MR-prioritised proteins and plaque vulnerability index using a linear model, with protein expression available for 1500 proteins and mRNA expression available from 55,105 transcripts, all measured in atherosclerotic plaques. We additionally performed subgroup analyses stratified by sex, age (<65 vs. ≥65 years), and diabetes status to assess the consistency of associations, and used formal interaction tests to evaluate differences across subgroups. Linearity assumptions of the linear models were evaluated using residuals versus fitted plots, which showed broadly linear patterns.^25^

### Determining cellular expression using single-cell RNA sequencing

Single-cell RNA sequencing was used to explore whether genes coding for MR-prioritised proteins were differentially expressed across plaque cell types, providing important information on the mechanisms driving atherosclerosis. A Wilcoxon rank-sum test was used to compare the expression in a single cell type to expression in the remaining cell types. The following cell types were considered: dendritic cells, endothelial cells I (ECs I), endothelial cells II (ECs II), foam cells, inflammatory macrophages, mast cells, memory B-cells, monocytes, natural killer cells (NK-cells), plasma B-cells, resident macrophages, smooth muscle cells (SMCs), and T-cells. Additionally, differential expression across broader clusters of cell types (structural cells, innate immune cells, and adaptive immune cells) was assessed using the Wilcoxon rank-sum test (Supplemental Methods).

### Annotations, effect estimates and multiple testing

Proteins are referred to using their Uniprot label^26^ and genes are referred to using the Ensembl label in italicised font. MR results are presented as mean differences (MD) for urinary metabolism breakdown products, or odds ratios (OR) for CHD, representing the effect of one standard deviation (SD) increase of the exposure (i.e., either urinary metabolism breakdown products or plasma protein value). Associations of the plaque vulnerability index with protein expression levels or mRNA expression levels are presented as MD in normalised count per unit increase in the plaque vulnerability index. All effect estimates are accompanied by 95% confidence intervals (CIs) and p-values. Metabolism breakdown product effects on CHD were filtered for a multiplicity corrected p-value threshold of 1.37 × 10^−4^ (0.05/365 = 1.37 × 10^−4^) based on the 365 principal components that were needed to explain 90% of the variance in the urinary metabolism breakdown products (Appendix Figure S1). Protein MR effect estimates of the metabolism breakdown product association were evaluated against a multiplicity corrected p-value threshold of 1.10 × 10^−6^ (0.05/(1562 × 29) = 1.10 × 10^−6^) based on the number of tested proteins (n = 1562) and metabolism breakdown products (n = 29). The multiplicity corrected p-value threshold was 7.33 × 10^−6^ (0.05/682 = 7.33 × 10^−6^) for the MRs of protein on CHD based on the number of tested proteins (n = 682). As the MR analyses involved large-scale hypothesis-free testing, we applied Bonferroni correction to control the family-wise error rate and minimise the chance of false positives. For analyses of associations of protein and mRNA expression levels with plaque vulnerability, p-values were adjusted using the Benjamini-Hochberg method^27^ with a false discovery rate threshold of 0.1. This method was appropriate for the more targeted follow-up analysis of MR-prioritised proteins in a smaller sample, where preserving statistical power was important while still accounting for multiple testing. For differential expression testing using single-cell RNA sequencing data, we used a nominal p-value threshold of 0.05.

### Druggability of prioritised proteins

We identified plasma proteins targeted by approved drugs (drugged proteins), as well as plasma proteins targeted by a developmental drug (druggable proteins) based on ChEMBL. For the drugged and druggable proteins, compound indications and side-effects were extracted from ChEMBL and the British National Formulary (BNF).

### Pathway enrichment analyses

To better understand the biological relevance of the identified urinary metabolism breakdown products, we performed a metabolite set enrichment analysis using MetaboAnalyst. Over-representation analysis was conducted against a reference set of known urine metabolites to identify enriched metabolic conditions and pathways. Similarly, to annotate the prioritised proteins, we performed pathway enrichment analysis using the Reactome knowledgebase-v85. All proteins available in the considered GWAS were mapped to biological pathways, and prioritised proteins were tested for enrichment against the full set of 1562 proteins.

### Partial replication of protein associations with cardiac traits

Due to the availability of eight proteomics GWAS, some studies measured the same proteins, which we used to replicate the associations with CHD risk. Replication was sought using a nominally significant p-value of 0.05 or smaller and considering the effect direction of the triangulated analysis. In addition, a more stringent p-value threshold of 6.94 × 10^−4^ (0.05 divided by the number of proteins that were available in more than one study) was used for more conservative replication.

### Ethics

The AE Biobank study is in line with the Declaration of Helsinki, and informed consent was provided by all study participants. Ethical approval was obtained from the Medical Research Ethics Committee of the University Medical Centre Utrecht (reference TME/C-01.18) and from the ethics committee of the St. Antonius Hospital, Nieuwegein, The Netherlands.

### Role of funders

The funding source did not influence the study design, the collection, analysis, and interpretation of data, the writing of the report, or the decision to submit the manuscript for publication.

## Results

### Urinary metabolism breakdown products associating with CHD

**Fig. 3:**
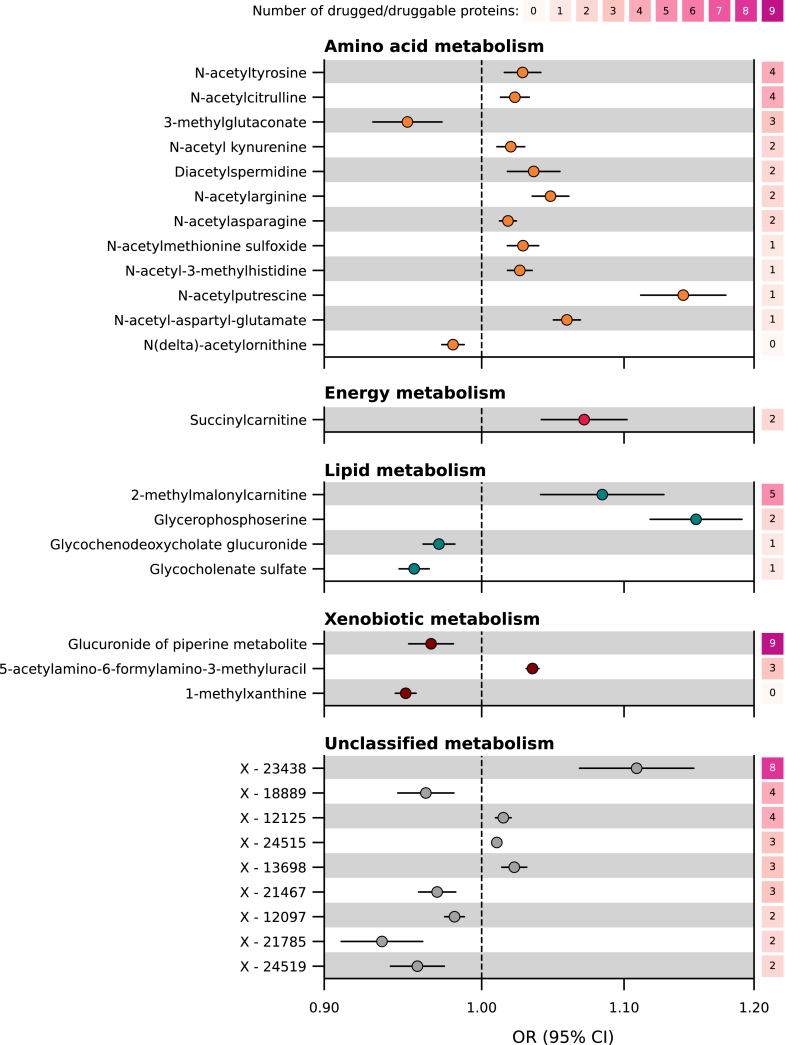
**Associations of urinary metabolism breakdown products with CHD, presented per metabolism class.** N.B. Point estimates represent associations between urinary metabolism breakdown products and coronary heart disease (CHD), obtained from genome-wide Mendelian randomisation. Horizontal lines represent 95% confidence intervals (CIs) for the odds ratios (ORs). The metabolism classes (amino acid, energy, lipid, xenobiotics, and unclassified) represent the metabolic origins of the breakdown product. The right y-axis indicates the number of drugged and druggable proteins associated with the metabolism breakdown product and CHD, where druggability is sourced from the British National Formulary and ChEMBL. Genetic associations with 954 urinary metabolism breakdown products were obtained from Schlosser et al. (n = 1627).^13^ Genetic associations with CHD were obtained from Aragam et al. (181,522 CHD cases).^14^ For more detailed information, please refer to the Methods section and Appendix Tables S1 and S2. Abbreviations: CI = confidence interval, OR = odds ratio.

Using Mendelian randomisation, we evaluated 954 urinary metabolism breakdown products to investigate their association with CHD (Appendix Table S1). Out of these, 29 were associated with CHD (Appendix Table S2, step 1 in Figs. 1 and 3). Twelve of these metabolism breakdown products originated from amino acid metabolism. For example, higher values of N-acetyltyrosine increased the risk of CHD (OR 1.03 per one standard deviation increase, 95% CI 1.02; 1.04), while higher values of 3-methylglutaconate decreased the risk of CHD (OR 0.95 per one standard deviation increase, 95% CI 0.93; 0.97). Other associated urinary metabolism breakdown products originate from various metabolic processes: one from energy metabolism, four from lipid metabolism, and three from xenobiotic metabolism. Finally, nine urinary metabolism breakdown products of unclassified metabolic origin were associated with CHD, indicating metabolic pathways that have not yet been characterised.

### Triangulated proteins concordant with urinary metabolism breakdown products and CHD

We used Mendelian randomisation to determine associations between plasma proteins and the 29 urinary metabolism breakdown products identified as being associated with CHD. We identified 682 proteins associating with at least one of these 29 urinary metabolism breakdown products (Appendix Figure S2). Among these, 113 proteins were identified with a directionally concordant association with CHD (steps 2–4 in Fig. 1, Appendix Figure S3 and Appendix Table S3). Please see, Supplemental Results and Appendix Tables S4–S5 for MR analyses replicating 83% of these findings in smaller independent GWAS.

### MR-prioritised proteins and plaque vulnerability index

**Fig. 4:**
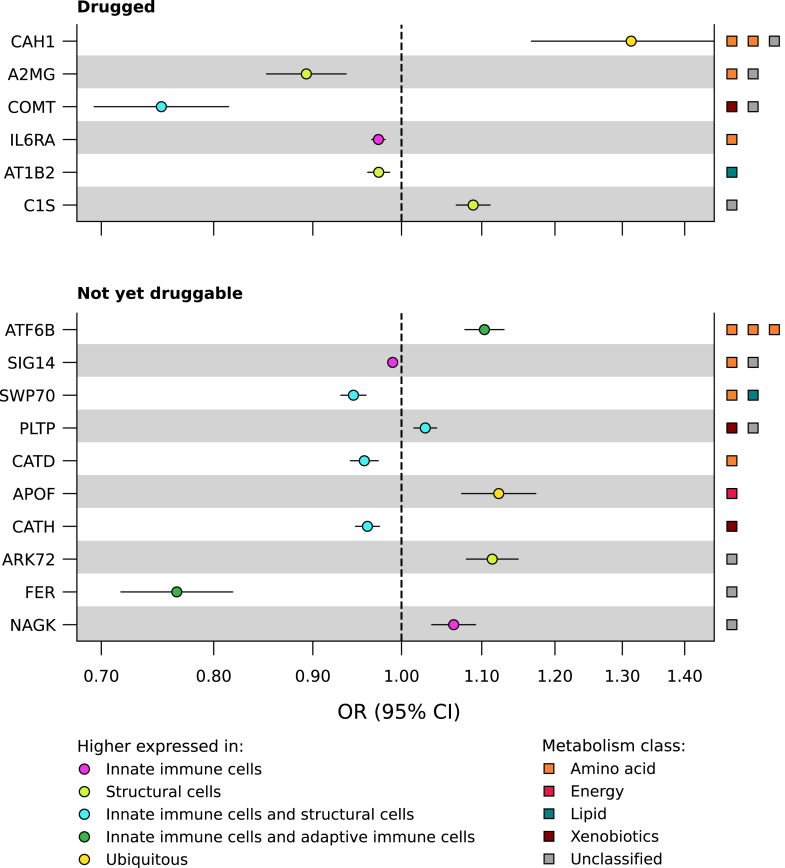
**Associations of prioritised proteins with CHD and plaque vulnerability in atherosclerotic plaque.** N.B. Point estimates represent associations between plasma proteins and coronary heart disease (CHD), obtained from *cis* Mendelian randomisation. Horizontal lines represent 95% confidence intervals (CIs) for the odds ratios (ORs). Druggability is based on ChEMBL and the British National Formulary, distinguishing between proteins targeted by approved drug (drugged proteins, top panel), proteins targeted by a drug in development (druggable proteins, middle panel) and not yet druggable proteins (bottom panel). Differential expression of genes coding for prioritised proteins is determined across cell types based on single-cell RNA sequencing data obtained from patients participating in the Athero-Express Biobank (4948 cells, 46 patients). The metabolism classes of the metabolism breakdown products affected by the protein are indicated on the right y-axis. Genetic associations with CHD were obtained from Aragam et al. (181,522 CHD cases).^14^ For a more detailed description, please refer to the Methods section and the Supplemental Methods. Full numerical results can be found in Appendix Table S3. Abbreviations: CI = confidence interval, OR = odds ratio.

The MR-prioritised proteins were further validated using the AE Biobank (a schematic overview is provided in Appendix Figure S4). Of the 113 MR-prioritised proteins, 36 were available and detectable in the AE protein expression data measured in plaque samples (194 patients, Table 1 for characteristics) and 10 proteins were associated with increased plaque vulnerability: A2M, AKR7A2, APOF, C1S, CA1, COMT, CTSD, CTSH, NAGK, and PLTP (Appendix Figure S5, Appendix Table S6). Data on mRNA expression in plaques was available for 110 genes coding for MR-prioritised proteins (632 patients, Table 1 for characteristics), identifying an additional 6 proteins where mRNA expression was associated with plaque vulnerability: FER, IL6RA, ATF6B, AT1B2, SWP70, and SIG14 (Fig. 4, Appendix Figure S6, Appendix Table S7).

We also assessed whether the associations between protein or mRNA expression and plaque vulnerability were consistent across clinical subgroups. We performed stratified analyses by sex, age (<65 vs. ≥65 years), and diabetes status. Among the protein associations, we observed a stronger association between PLTP expression and plaque vulnerability in women compared to men. No other subgroup differences were observed in either the protein or mRNA analyses (Appendix Tables S8–S9).

### Single-cell RNA sequencing of MR-prioritised proteins

**Fig. 5:**
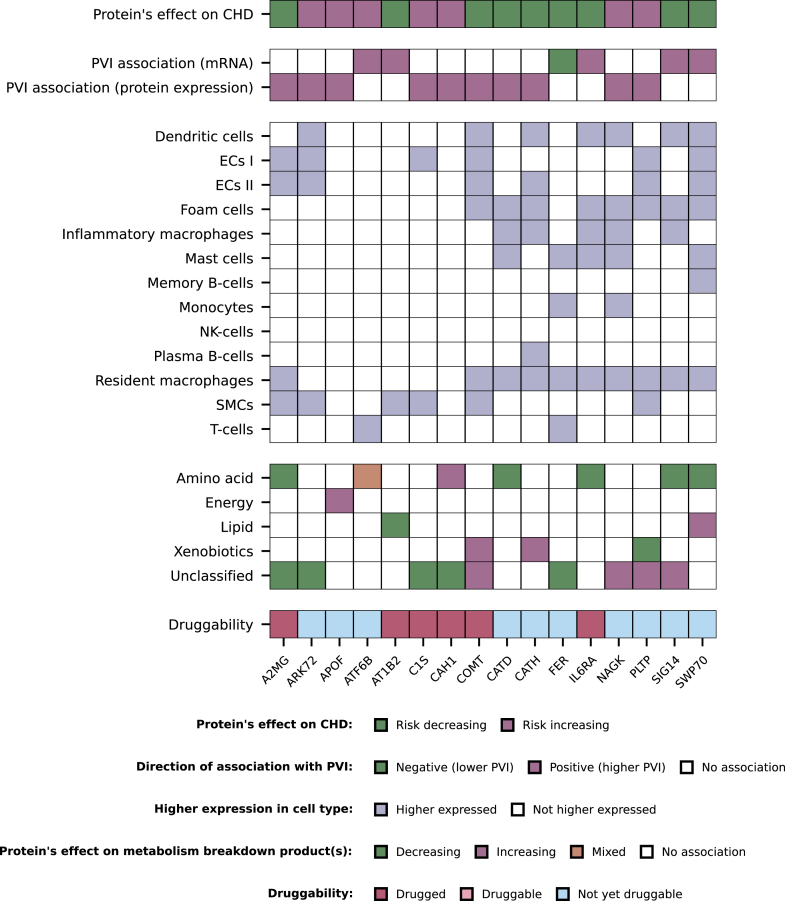
**Matrix of prioritised proteins associated with CHD and plaque vulnerability in atherosclerotic plaque.** N.B. Each column represents a prioritised protein associated with coronary heart disease (CHD) and plaque vulnerability. The gene names corresponding to each protein are presented in Table 2. The rows present the following: row 1, direction of the protein's association with CHD as obtained by Mendelian randomisation; row 2–3, association of the gene's mRNA or protein expression in plaque with plaque vulnerability, obtained from the Athero-Express Biobank; row 4–16, higher expression in a plaque cell type as compared to expression in all other plaque cell types, obtained from single-cell RNA sequencing in the Athero-Express Biobank; row 17–21, direction of the protein's association with metabolism breakdown product(s) presented per metabolism class, based on Mendelian randomisation; row 22, druggability status, distinguishing proteins targeted by approved drug (drugged proteins) from proteins targeted by a drug in development (druggable proteins) and not yet druggable proteins, based on ChEMBL and the British National Formulary. Full numerical results can be found in Appendix Tables S5, S6–S8. Abbreviations: CHD = coronary heart disease, ECs = endothelial cells, NK-Cells = natural killer cells, PVI = plaque vulnerability index, SMCs = smooth muscle cells.

To understand the cellular origin of the proteins, we additionally explored which genes encoding prioritised proteins were differentially expressed across cells in carotid plaques. For this, we used single-cell RNA sequencing data on over 4900 cells available for 105 of our MR-prioritised proteins including all 16 proteins associated with plaque vulnerability. We identified 87 genes that showed to be enriched in one or more plaque cell types, which included 14 genes encoding for the proteins associated with plaque vulnerability (Appendix Table S10, Fig. 5). For example, the gene *ATP1B2*, coding the protein AT1B2, was higher expressed in smooth muscle cells as compared to other plaque cell types (Fig. 5, Appendix Figure S7, Appendix Table S10). The gene *ATF6B*, coding for the protein ATF6B, was higher expressed in T-cells as compared to other plaque cell types. Next, we used broader clusters of cell types, namely structural cells, innate immune cells, and adaptive immune cells. We found three (IL6RA, SIG14, and NAGK) out of 16 plaque vulnerability-associated proteins to be higher expressed in innate immune cells as compared to the other two clusters of cell types, while four (AT1B2, C1S, A2MG, and ARK72) were higher expressed in structural cells, five (COMT, CATD, CATH, PLTP, and SWP70) were higher expressed in both innate immune and structural cells and two (FER and ATF6B) were higher expressed in both innate immune cells and adaptive immune cells (Fig. 4). Two proteins (CAH1 and APOF) were ubiquitous (i.e., no higher expression in any of the three clusters).

### Druggability of proteins

The set of 16 proteins associated with plaque vulnerability included six drugged proteins and ten proteins which are not yet druggable (step 6 in Figs. 1, 4 and 5). These proteins predominantly associated with amino acid breakdown products and unclassified breakdown products. Four drugged proteins (AT1B2, CAH1, COMT, and IL6RA) were targeted by a drug with a cardiac indication and/or side-effect (Table 2, Fig. 4, Appendix Tables S11 and S12). No metabolic pathways were overrepresented in our set of prioritised breakdown products, but we did observe partial overlap of some breakdown products with conditions related to amino acid catabolism, nitric oxide synthesis, and energy buffering (results of enrichment analysis can be found in Appendix Table S13). The protein AT1B2 (targeted as part of a protein complex group through digoxin which is contraindicated in HCM) decreased the risk of CHD (OR 0.97, 95% CI 0.96; 0.99). Higher values of CAH1 (targeted by methocarbamol which has registered side-effects including blood pressure disorders) increased the risk of CHD (OR 1.31, 95% CI 1.17; 1.48). Higher values of COMT (targeted by the drugs tolcapone and entacapone belonging to the class of COMT-inhibitors indicated for Parkinson's disease which have side-effects including chest pain and increased risk of CHD) decreased the risk of CHD (OR 0.75, 95% CI 0.69; 0.81). Higher values of IL6RA, interleukin-6 receptor subunit alpha, decreased the risk of CHD (OR 0.97, 95% CI 0.97; 0.98). IL6RA is targeted by the interleukin-6 receptor antagonist tocilizumab indicated for auto-immune diseases, which is also in phase 2 development for CHD^28^ and has registered side-effects of blood pressure disorders.

**Table 2:** Positive controls and repurposing opportunities: prioritised proteins related to plaque vulnerability.

Protein	Gene name	Uniprot ID	Druggability	Metabolism class(es)	No. breakdown products	Breakdown product–CHD effect	Protein-CHD effect	No. drugs	Cardiac indication and/or side-effect	Cardiac indication(s)	Cardiac side–effect(s)	Drug name(s)
A2MG	A2M	P01023	Druggable	Unclassified, Amino Acid	2	Increasing	0.89 (0.85; 0.94)	1	No	–	–	Technetium tc 99m succimer
ARK72	AKR7A2	O43488	Not yet druggable	Unclassified	1	Decreasing	1.11 (1.08; 1.15)	0	No	–	–	
APOF	APOF	Q13790	Not yet druggable	Energy	1	Increasing	1.12 (1.07; 1.17)	0	No	–	–	
ATF6B	ATF6B	Q99941	Not yet druggable	Amino Acid	3	Mixed	1.10 (1.08; 1.13)	0	No	–	–	
AT1B2	ATP1B2	P14415	Drugged	Lipid	1	Increasing	0.97 (0.96; 0.99)	6	Yes	See Appendix Table S11	Arrhythmias, Cardiac conduction disorder	Deslanoside, Digitoxin, Digoxin, Acetyldigitoxin, Istaroxime, Lanatoside c
C1S	C1S	P09871	Drugged	Unclassified	1	Decreasing	1.09 (1.07; 1.11)	2	No	–	–	Human c1-esterase inhibitor, Sutimlimab
CAH1	CA1	P00915	Drugged	Unclassified, Amino Acid	3	Mixed	1.31 (1.17; 1.48)	8	Yes	Pulmonary hypertension, Heart failure	Arrhythmias, Blood pressure disorders	Methocarbamol, Acetazolamide sodium, Dichlorphenamide, Acetazolamide, Methazolamide, Polmacoxib, Ethoxzolamide, Sulthiame
COMT	COMT	P21964	Drugged	Xenobiotics, Unclassified	2	Decreasing	0.75 (0.69; 0.81)	4	Yes	–	Myocardial infarction, Chest pain	Tolcapone, Entacapone, Opicapone, Nebicapone
CATD	CTSD	P07339	Not yet druggable	Amino Acid	1	Increasing	0.96 (0.94; 0.97)	0	No	–	–	
CATH	CTSH	P09668	Not yet druggable	Xenobiotics	1	Decreasing	0.96 (0.95; 0.97)	0	No	–	–	
FER	FER	P16591	Not yet druggable	Unclassified	1	Increasing	0.77 (0.72; 0.82)	0	No	–	–	
IL6RA	IL6R	P08887	Drugged	Amino Acid	1	Increasing	0.97 (0.97; 0.98)	5	Yes	Non-st elevated myocardial infarction, Pulmonary hypertension	Blood pressure disorders	Tocilizumab, Sarilumab, Vobarilizumab, Levilimab, Satralizumab
NAGK	NAGK	Q9UJ70	Not yet druggable	Unclassified	1	Increasing	1.06 (1.04; 1.09)	0	No	–	–	
PLTP	PLTP	P55058	Not yet druggable	Unclassified, Xenobiotics	2	Mixed	1.03 (1.01; 1.04)	0	No	–	–	
SIG14	SIGLEC14	Q08ET2	Not yet druggable	Unclassified, Amino Acid	2	Mixed	0.99 (0.98; 0.99)	0	No	–	–	
SWP70	SWAP70	Q9UH65	Not yet druggable	Amino Acid, Lipid	2	Mixed	0.94 (0.93; 0.96)	0	No	–	–	

N.B. Each row presents a plasma protein associated with coronary heart disease (CHD), and at least one urinary metabolism breakdown product, where the associations between protein, breakdown product(s), and CHD are directionally concordant (Fig. 2). Associations were identified by Mendelian randomisation.

Protein names are based on their Uniprot synonym. Druggability indicates whether the proteins are targeted by a developmental compound in clinical trials (druggable) or by a compound which has received marketing approval (drugged), based on ChEMBL and the British National Formulary. The metabolism classes represent the identified metabolism origin of the urinary breakdown product. The breakdown product–CHD effect indicates whether the breakdown product's effect on CHD is risk-increasing or risk-decreasing, based on *cis-*Mendelian randomisation. The protein–CHD effect estimates were obtained from *cis*-Mendelian randomisation and are presented as odds ratios representing the effect of a one standard deviation increase in protein values, along with corresponding confidence intervals. The No. drug column records the number of approved drugs with affinity for the protein as available in ChEMBL. For more details on metabolism breakdown products and full numerical Mendelian randomisation results please refer to Appendix Table S3. For a full list of the compounds and their indication and side-effects please refer to Appendix Table S11.

**Fig. 6:**
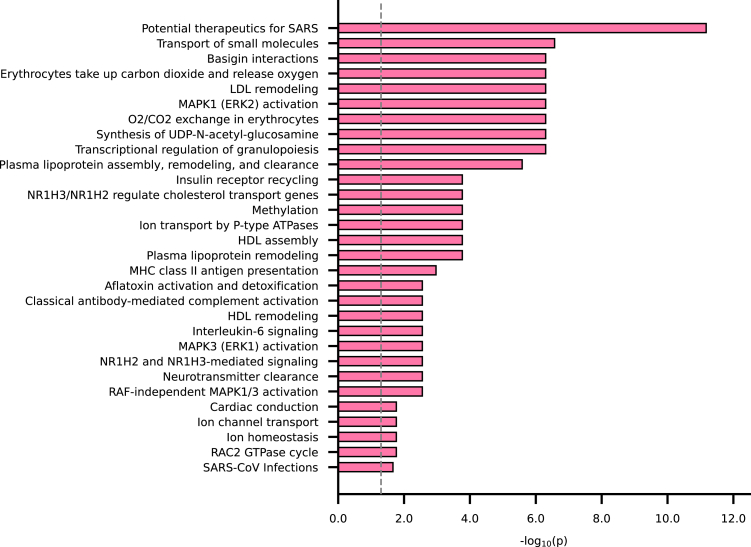
Reactome pathways and the corresponding enrichment p-value. N.B. Enrichment was based on the Reactome knowledgebase-v85. Prioritised proteins associated with plaque vulnerability were tested for enrichment against the full set of 1562 proteins. P-values were adjusted for multiple testing using the Benjamini-Hochberg false discovery procedure. The significance threshold of FDR 0.05 is indicated as a vertical line.

Based on the Reactome knowledgebase, we found several pathways enriched among the prioritised proteins associated with PVI, which involved lipid metabolism (e.g., HDL remodelling, NR1H2/3 signalling), inflammatory signalling (e.g. IL-6, MAPK1/3), and immune-related processes (e.g. MHC class II presentation, complement activation). These results highlight the multifactorial aetiology of atherosclerosis. All enriched pathways are shown in Fig. 6, with full details of Reactome pathway enrichment in Appendix Table S14.

## Discussion

In this study, we used Mendelian randomisation to assess relationships between 954 urinary metabolism breakdown products, 1562 proteins and CHD, and we linked protein and mRNA expression levels in carotid plaque to plaque vulnerability. This was done to identify potential biomarkers of altered metabolism informative of CHD risk and plasma proteins which may be used as targets for intervention in these metabolic pathways. Through our multi-layered approach, we prioritised 113 plasma proteins associated with CHD risk, of which 83% were replicated in independent proteomics GWAS using multiple platforms, supporting the robustness of the genetic associations. Among these, 16 proteins also showed associations with plaque vulnerability. The majority of these proteins were associated with urinary metabolism breakdown products from amino acid metabolism or unclassified origins.

Altered amino acid metabolism in cardiovascular disease has been observed before.^29^, ^30^, ^31^, ^32^ For example, the kynurenine pathway has been linked to the pathogenesis of atherosclerosis by modulating inflammation, oxidative stress, and endothelial function.^33^ Its breakdown products were identified as biomarkers of major cardiovascular events,^34^^,^^35^ with previous studies showing the therapeutic potential of targeting this pathway to target vascular inflammation and plaque formation.^36^ We were able to link N-acetylkynurenine to the proteins SIG14 and A2MG. SIG14 is member of the sialic acid-binding immunoglobulin-type lectins (Siglecs) family, which is involved in immune regulation by promoting NLRP3 inflammasome activation and subsequent IL-1β release and has been studied as drug target for various diseases including asthma, cancer, and autoimmune diseases.^37^^,^^38^ A2MG is a broad-spectrum protease inhibitor with an anti-inflammatory role and it has a dual role in coagulation,^39^^,^^40^ which we now link to plaque vulnerability. By inhibiting enzymes such as thrombin, plasmin, and kallikrein, A2MG can influence fibrinolytic balance and promote a pro- or antithrombotic state depending on context.

The 16 prioritised proteins included one protein with a known indication or side-effect on CHD, namely COMT. COMT is affected by various drugs, including the Parkinson's disease drugs entacapone and tolcapone which is known to increase the risk of myocardial infarction through effects on blood pressure, arteriosclerosis, and aortic stenosis.^41^

This positive control finding confirmed our analysis was able to identify known drug target effects for CHD. Additionally, we identified two drugged proteins (AT1B2 and IL6RA) which are in development for CHD or CHD-related diseases. IL6R inhibiting drugs (e.g., tocilizumab) are currently being repurposed for treatment of CHD,^9^^,^^12^ where the phase 3 CANTOS trial evaluating canakinumab, inhibiting IL-1 which is upstream of IL6 and IL6R, showed clinical effectiveness in preventing CHD.^42^ This provides additional support for our approach as we rediscovered known drug target effects for CHD.

The drugged protein C1S was identified as potential repurposing candidates for treatment of CHD and atherosclerosis. C1S is inhibited by drugs such as the human c1-esterase inhibitor indicated for conditions such as angioedema. C1S activates the classical pathway of the complement system, contributing to inflammation and endothelial dysfunction, which promotes atherosclerosis.^43^^,^^44^ The here identified effects of higher values of C1S on increased CHD risk, and association with increased plaque vulnerability, along with higher expression in smooth muscle cells and endothelial cells in plaque, supports drug repurposing for management of CHD and atherosclerosis.

We additionally identified ten not yet druggable proteins (ARK72, APOF, ATF6B, CATD, CATH, FER, SWP70, NAGK, PLTP, and SIG14) with associations with CHD and plaque vulnerability. Higher values of APOF (apolipoprotein F) were associated with an increased CHD risk and its expression was higher in plaques with higher vulnerability. Previous studies have described APOF as a potential target for therapeutic approaches.^45^ APOF regulates CETP activity, affecting the transfer of cholesteryl esters from high density lipoprotein (HDL) to low density lipoprotein (LDL), which is known to affect HDL and LDL cholesterol plasma concentrations and atherosclerosis risk.^46^^,^^47^ We have previously shown that despite many failed attempts to develop sufficiently potent and selective compounds targeting CETP, the drug target itself remains a viable target for preventing CHD supporting the ongoing phase 3 study of obicetrapib.^11^^,^^48^^,^^49^

Other not yet druggable proteins we identified have plausible mechanistic links to CHD as well. NAGK is involved in amino sugar metabolism and plays a role in atherosclerosis through endothelial cell activation and inflammatory responses. Glucosamine, a substrate of NAGK, has been shown to suppress endothelial cell activation via O-GlcNAc modification, thereby potentially reducing atherosclerotic inflammation.^50^ By identifying an association of higher values of NAGK with a higher CHD risk and plaque vulnerability, the current study provides further support that inhibiting NAGK may be a viable strategy to treat CHD and atherosclerosis. ARK72 detoxifies lipid peroxidation-derived aldehydes and may protect against oxidative stress-driven endothelial injury, a key process in early atherogenesis.^51^ ATF6B is a regulator of the endoplasmic reticulum unfolded protein response, which modulates proteostasis and inflammatory signalling in vascular cells under stress, both of which are implicated in plaque formation and progression.^52^ SWP70 (SWAP70) controls actin cytoskeleton remodelling and MHC II trafficking in immune cells, influencing macrophage activation within plaques.^53^ PLTP (phospholipid transfer protein) plays a key role in HDL remodelling and lipid transport, processes critical for foam cell formation and plaque lipid content.^54^^,^^55^ FER is a non-receptor tyrosine kinase involved in cytoskeletal organisation and leucocyte–endothelial interactions, potentially affecting inflammatory cell recruitment to lesions. CTSH (cathepsin H) and CATD (cathepsin D) belong to the same cysteine protease family; these enzymes contribute to ECM remodelling, inflammation, and T-cell activation in plaques.^56^^,^^57^

Identifying MR-prioritised proteins which are overexpressed in specific plaque cell types advances the understanding of their potential roles in atherosclerosis. For example, proteins enriched in innate immune cells (e.g., IL6RA, SIG14, NAGK) may contribute to inflammatory signalling, and proteins enriched in smooth muscle cells (e.g., AT1B2, A2MG) may be related to plaque stability and vascular remodelling. These insights help clarify how the targets may be used in therapeutic strategies.

Despite the robustness of our analyses, our study has a number of potential limitations. Firstly, the GWAS data we sourced used various high throughput assays, varying in accuracy and analytic scope.^58^ These assays do not directly measure concentrations of proteins or metabolism breakdown products, but evaluate relative values. Therefore, the magnitude of associations is unlikely to be a strong indicator of the effects of pharmacological perturbation of the target, and smaller MR effects may translate towards large drug effects and vice versa. Instead, these results predominantly provide information on the drug mechanisms and whether targets should be inhibited or activated. In the current study, urinary metabolism breakdown products were used as a proxy to identify more distal metabolic processes occurring in, or impacting, atherosclerotic relevant tissues. These breakdown products are metabolites that have already been excreted. Hence, the associations between urinary metabolism breakdown products and CHD do not reflect a direct causal mechanism but rather point to the upstream metabolism origins of these breakdown products, which may play an important role in the development of atherosclerosis and the risk of CHD. Unlike plasma metabolites, which reflect circulating compounds anticipated to be more proximal to atherosclerosis processes, urinary metabolism breakdown products may capture more distal activity shaped by renal clearance and excretion which reflects systemic metabolic changes known to contribute to pathogenesis.^59^ For some of the MR-prioritised proteins linked with plaque vulnerability, we found an opposite direction of the association of the protein expression in plaque with the plaque vulnerability as compared to the effect of the circulating protein on CHD risk. These findings underscore the complexity of tissue-specific expression, and the molecular pathways involved in atherogenesis. Protein expression patterns can vary by tissue type and stage of atherosclerosis,^60^^,^^61^ with certain proteins possibly being present due to cell death rather than active secretion, which may affect the interpretation of their direction of effect. Furthermore, MR-prioritised plasma proteins may be involved in CHD through mechanisms in other tissues, such as arterial constriction, without accumulating in plaques. As a result, these proteins may not be represented in our AE findings. However, since plaques do no synthesise proteins but contain proteins originating from plasma, the plaque proteins we identified are likely linked to the plasma proteins prioritised by MR as being related to CHD. Both the GWAS and the AE Biobank data we used predominantly included European individuals. This limits the generalisability of our findings to non-European populations due to differences in genetic architecture, environmental exposures, or clinical characteristics. Future studies are required to evaluate these findings in more diverse cohorts to assess whether the identified therapeutic targets are relevant across populations. In addition, while the source GWAS included both men and women, they did not provide aggregated results stratified by sex and hence we were unable to assess sex-differential effects in the Mendelian randomisation analyses. Lastly, the observational nature of the AE and its high-risk study population introduces a risk of bias due to for example confounding, reverse causation, and index event bias. However, combining these analyses with MR, which is largely confounding-resistant, meaningfully enhances the robustness of our findings.

In conclusion, we identified urinary biomarkers of altered metabolism, predominantly from amino acid metabolism and unclassified origins, associating with CHD risk. By integrating these findings with plasma proteins and plaque vulnerability, we identified 16 plasma proteins as potential drug targets for CHD, supporting the development of (repurposed) therapeutic strategies. Additionally, our study highlights the value of integrating multi-modal evidence to uncover information potentially relevant for disease diagnosis, prognosis, and aetiology.

## Contributors

SR, EDB, AFS, and SP designed the study. SR and MV accessed the data, verified the data, and performed analyses. SR drafted the manuscript. SR, EDB, AFS, SP, MV, CF, DG, DK, GP, and HR provided critical input on the analysis, as well as the drafted manuscript. SR, EDB, AFS, SP, MV, CF, DG, DK, GP, and HR had full access to all the data in the study, accept responsibility to submit for publication, and read and approved the final version of the manuscript.

## Data sharing statement

The GWAS data on CHD used in this study can be accessed via https://www.ebi.ac.uk/gwas/studies/GCST90132314 (n = 181,522 CHD cases, n = 984,168 controls). The GWAS data on urinary metabolism breakdown product values can be accessed via https://www.ebi.ac.uk/gwas/publications/31959995 (n = 1627). The individual GWAS data on plasma protein values can be accessed as follows: deCODE (n = 35,559, https://www.decode.com/summarydata/), SCALLOP (n = 30,931, https://www.ebi.ac.uk/gwas/publications/33067605), Ahola-Olli et al. (n = 8,293, https://www.ebi.ac.uk/gwas/publications/27989323), Framingham (n = 6,861, https://www.ebi.ac.uk/gwas/publications/21909115), AGES-Reykjavik (n = 5,368, https://www.ebi.ac.uk/gwas/publications/35078996), INTERVAL (n = 3,301, https://www.ebi.ac.uk/gwas/publications/29875488), Gilly et al. (n = 1,328, https://www.ebi.ac.uk/gwas/publications/33303764), and Yang et al. (n = 636, https://www.ebi.ac.uk/gwas/publications/34239129). The data and code used in this study is available through GitLab https://gitlab.com/SophiedeRuiter/mr-project-chd as well as on Figshare https://doi.org/10.5522/04/30178309. The Athero-Express data are upon request and conditional on GDPR and ethical approvals from Ernest Diez Benavente: e.diezbenavente@umcutrecht.nl.

## Code availability

Analyses were conducted using Python v3.7.13 (for GNU Linux), Pandas v1.3.5, Numpy v1.21.6, bio-misc v0.1.4, plot-misc, matplotlib v3.4.3, and merit (to implement GLS-IVW, MR-Egger, and model selection procedures). Single-cell RNA sequencing data were analysed in R using Seurat v3.^62^ The code has been made available through GitLab https://gitlab.com/SophiedeRuiter/mr-project-chd and Figshare https://doi.org/10.5522/04/30178309.

## Declaration of interests

AFS and CF have received funding from New Amsterdam Pharma for unrelated projects. The other authors declare that they have no conflict of interest.
